# A Rare p.T599dup BRAF Mutant NSCLC in a Non-Smoker

**DOI:** 10.3390/curroncol28010021

**Published:** 2020-12-25

**Authors:** Alla Turshudzhyan, James Vredenburgh

**Affiliations:** 1 University of Connecticut, Internal Medicine, Farmington, CT 06030, USA; 2 Hematology and Oncology Department Saint Francis Hospital, Hartford, CT 06105, USA; jvredenb@stfranciscare.org

**Keywords:** BRAF, non-V600, pT599dup, NSCLC

## Abstract

V-RAF murine sarcoma viral oncogene homolog B1 (BRAF) mutated non-small-cell lung cancer (NSCLC) is an exceptionally rare form of lung cancer, found only in one to two percent of patients with an NSCLC diagnosis. BRAF NSCLC traditionally affects former or active smokers. BRAF mutations have always been of special interest to the oncological community, as they offer potential for targeted therapies. BRAF mutation spectrum includes mutations that are of both V600 and non-V600 types. BRAF V600 is an activating mutation, which results in high kinase activity and overproduction of active oncoproteins such as rapidly accelerated fibrosarcoma (RAF). This makes them susceptible to targeted therapies with RAF inhibitors. There has been little evidence, however, regarding efficacy of RAF inhibitors towards non-activating mutations that have intermediate to low kinase activity, such as non-V600 BRAF mutations. While several approaches have been investigated to overcome the limitations of RAF inhibitors, such as use of mitogen-activated protein kinase kinase (MEK) and extracellular signal-regulated kinase (ERK) inhibitors or combination of MEK and RAF inhibitors, none of them have been proven to have a superior efficacy for low kinase activity non-V600 BRAF tumors. We present a case of an extremely rare variant of NSCLC BRAF p.T599dup mutation in a non-smoker that responded to a targeted combination therapy with RAF and MEK inhibitors. The patient responded well to therapy that usually targets high kinase activity V600 mutations. Our hope is to bring more attention to non-V600 mutations and document their responses to existing and new therapies.

## 1. Introduction

V-RAF murine sarcoma viral oncogene homolog B1 (BRAF) mutation frequently seen in cancers causes deregulation of the mitogen-activated protein kinase (MAPK) pathway [1]. It most commonly occurs at V600 and results in predominantly active oncoproteins that lead to uncontrolled proliferation of cells and eventually malignancy [1,2]. BRAF V600 mutations are primarily seen in malignant melanomas but can also be found in thyroid, colorectal, and lung cancers [1]. Non-V600 BRAF mutations, however, are largely seen in lung cancers and melanomas [1,3,4]. BRAF V600 is an activating mutation, which results in high kinase activity and overproduction of active oncoproteins, such as rapidly accelerated fibrosarcoma (RAF) [1,5]. This makes them susceptible to targeted therapies with RAF inhibitors [1,5]. There has been little evidence, however, regarding efficacy of RAF inhibitors towards non-activating mutations that have intermediate to low kinase activity, such as non-V600 BRAF mutations [1,5]. While several approaches have been investigated to overcome the limitations of RAF inhibitors, such as use of mitogen-activated protein kinase kinase (MEK) and extracellular signal-regulated kinase (ERK) inhibitors or combination of MEK and RAF inhibitors, none of them have been proven to have a superior efficacy for low kinase activity non-V600 BRAF tumors [1].

We would like to present a case of a BRAF mutated non-small cell lung cancer (NSCLC), an exceptionally rare form of lung cancer found only in one to two percent of patients carrying an NSCLC diagnosis and usually affecting patients who are former or current smokers [5,6]. The presented case illustrates an extremely rare variant of BRAF p.T599dup mutation in a non-smoker that responded to targeted combination therapy with RAF and MEK inhibitors.

## 2. Case Report

Informed Consent: Written informed consent was obtained from the patient for publication of this case report and accompanying images.

**Figure 1:**
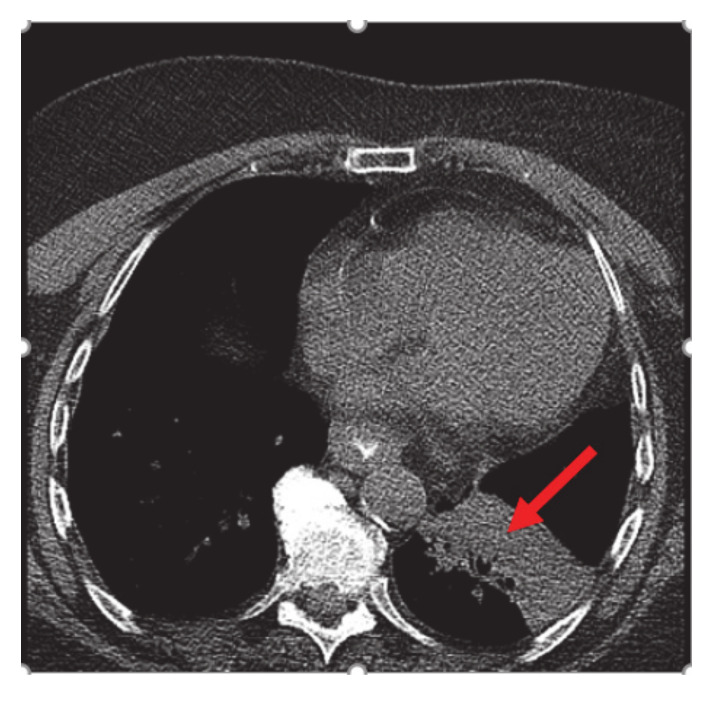
CT chest: left lower lobe consolidation/atelectasis (red arrow) with obscuration of proximal left lower lobe bronchus and mildly enlarged mediastinal nodes.

A 75-year-old non-smoker female with personal history of estrogen receptor (ER), progesterone receptor (PR) positive, Her2/neu negative breast cancer diagnosed 14 years ago, status post left breast lumpectomy, radiation, and 5 years of anastrozole with no evidence of recurrence, initially presented in early February 2020 for symptoms of persistent cough productive of green phlegm since January. She received azithromycin and prednisone for presumed pneumonia without improvement of symptoms. Later, she received levofloxacin and was referred to pulmonology for further evaluation. Computer tomography (CT) scan at that time showed left lower lobe consolidation/atelectasis with obscuration of proximal left lower lobe bronchus as well as mildly enlarged mediastinal nodes, concerning for underlying peribronchial lesion (Figure 1).

**Figure 2:**
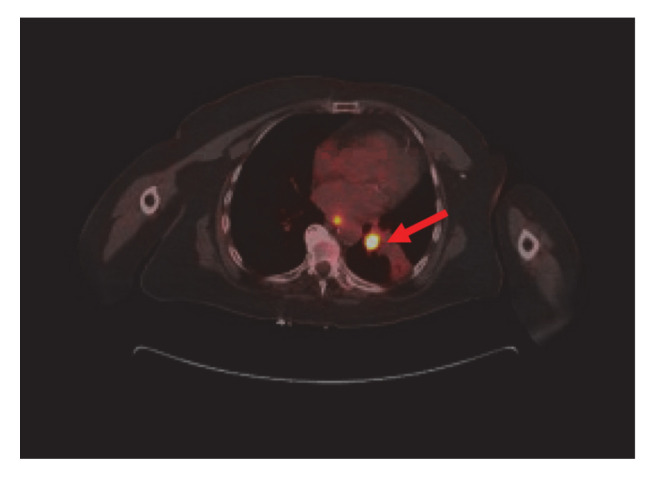
Nuclear Medicine Positron Emission Tomography/Computer Tomography (NM PET/CT): left lower lobe hypermetabolic neoplasm (red arrow) causing bronchial obstruction and distal collapse. Metastatic mediastinal adenopathy. Not shown, L3 vertebral body, left acetabulum pubis, and superior pubic ramus metastasis.

Bronchoscopy revealed endobronchial lesion involving the takeoff of the left lower lobe. PET scan showed left lower lobe hypermetabolic mass causing bronchial obstruction, distal collapse, and consolidation of basilar segments as well as subcarinal lymphadenopathy, osteolytic destruction of left acetabulum pubis, superior pubic ramus, and L3 vertebral body (Figure 2).

The patient was referred to oncology after endobronchial ultrasound bronchoscopy (EBUS) pathology returned as a non-small cell carcinoma compatible with adenocarcinoma of the lung. The tumor cells were positive for TTF-1 and negative for P 40. Patient’s mutation analysis revealed a BRAF mutation, specifically c.1794 1796 dup TAC (p.T599dup). Mutation analysis was done by isolating DNA from cells and evaluating the entire coding region of BRAF exon 15 for BRAF mutations by high-sensitivity Sanger sequencing and comparing patient’s sequence to the National Center for Biotechnology Information (NCBI) database NM_004333.

**Figure 3:**
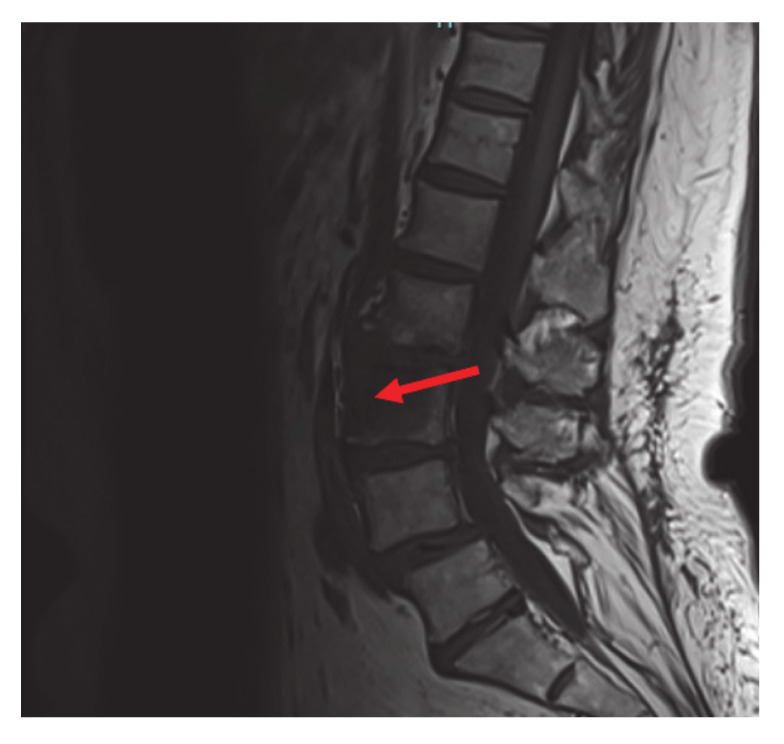
Magnetic Resonance Imaging (MRI) lumbar spine: L3 vertebral body metastasis (red arrow) without compression deformity or canal compromise. Not visualized, severe facet arthropathy with grade 1 anterolisthesis of L4 on L5.

The mutation detected is different from the more common V600 mutation and is an extremely rare variant of a non-V600 mutation. Non-V600 mutations are not usually seen in non-smokers, which makes this case more unusual. The patient was referred for palliative left hip and pelvic radiation with plans to start RAF and MEK inhibitors dabrafenib and trametinib after completion of radiation therapy. Guardant 360 confirmed the BRAF pT599dup mutation. Following completion of radiation to the hip, her pain was significantly improved, and she was started on dabrafenib/trametinib, only complaining of mild nausea that responded to anti-emetics. The patient also had an Magnetic Resonance Imaging (MRI) of cervical, thoracic, and lumbar spine done that showed an L3 vertebral body metastasis without compression deformity or canal compromise and severe facet arthropathy with grade one anterolisthesis of L4 on L5 (Figure 3).

**Figure 4:**
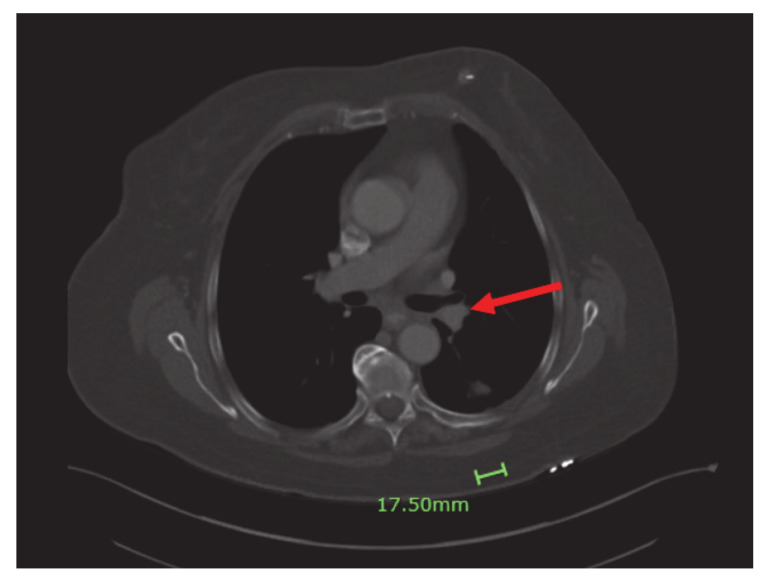
Significant regression of the left lower lobe bronchial tumor (red arrow) and re-expansion of left lower love.

Four months into the treatment, the patient had a repeat CT scan done, which showed marked regression of the left lower lobe bronchial tumor (Figure 4) and re-expansion of the left lower lobe. The patient had been responding well to dabrafenib and trametinib. She continued with both agents with minimal side effects.

## 3. Discussion

BRAF is a member of the RAF family of kinases, which mediate cell proliferation and are located downstream of rat sarcoma (RAS) in the RAS-RAF-MEK-ERK signaling pathway [7]. Once RAS is activated, BRAF phosphorylates a dual-specificity MEK, which leads to the activation of ERK and subsequently the ERK signaling pathway [7]. ERK signaling pathway has multiple negative regulatory events that serve to inhibit the pathway via negative feedback [8*]. Oncogenic mutations such as BRAF drive many cancers via ERK dependent growth by activating both downstream signaling as well as negative feedback mechanisms [8,9]. V600 is the most common BRAF mutations, but non-V600 mutations account for more than 50% of the BRAF mutations seen in lung cancers [8]. BRAF mutations can be further divided into activating mutations that cause feedback inhibition of GTP-bound RAS, are RAS-independent, and signal active monomers (class 1) or dimers (class 2) versus kinase-dead BRAF mutations that have little to no kinase activity (class 3) [10]. Class 3 mutants are sensitive to ERK-mediated feedback, and their signaling activation is RAS-dependent [10]. Kinase-dead BRAF mutations in lung and colorectal cancer are sensitive to the inhibition of RAS activation by inhibiting of the receptor tyrosine kinases [10]. While class 1 BRAF mutations are predominantly V600 and are sensitive to RAF monomer inhibitors, class 2 BRAF mutations, though still RAS-independent, are resistant to RAF inhibitors, as they activate dimers rather than monomers [10]. Class 3 BRAF mutations, on the other hand, being RAS-dependent, are sensitive to MEK inhibitors [10].

The development of the first-generation RAF inhibitors, such as vemurafenib and dabrafenib, led to initial breakthrough due to high response rate among malignancies carrying BRAF V600 mutations, seen predominantly in class 1 BRAF mutations [11]. This, however, was soon overshadowed by newly developing intrinsic and acquired drug-resistance mechanisms, such as previously described RAF dimerization, characteristic of class 2 BRAF mutations [11]. A more profound understanding of dimer interface (DIF) structure and how it impacts inhibitor activation in recent years facilitated development of the next generation of RAF inhibitors and DIF inhibitors [11]. BRAF mutations have shown response to targeted therapy with not only tyrosine kinase inhibitors such as RAF inhibitors, described earlier, but also with MEK inhibitors [12]. RAF/MEK inhibitors combination is dabrafenib and trametinib and is currently considered a first-line therapy for V600 subtypes of BRAF mutant cancers [13].

Ongoing development of targeted therapies for BRAF mutant cancers becomes even more important when it comes to BRAF mutated NSCLC presented in our case, as its clinical behavior tends to be more aggressive and resistant to chemotherapy [12]. The BRAF mutation described in the case, p.T599dup, results in a threonine insertion in place of the critical valine 600 and a subsequent shift by one codon. This affects the V600 region known to be modified in classical, inhibitor-sensitive activating mutations. We may thus hypothesize that the functional consequences of p.T599dup resemble those of p.V600 mutations. This is supported by the clinical finding of an objective response to RAF/MEK inhibitor therapy.

Treatment of cancers harboring non-V600 mutations is challenging due to functional heterogeneity, lack of knowledge of their clinical significance, or response to target therapies [14]. There has been one case reported, where a double non-V600 BRAF NSCLC responded well to RAF/MEK inhibition that usually targets V600 mutations [15]. Another case by Dagogo-Jack presented a durable response to dabrafenib combined with trametinib in a non-V600 (specifically G469A) mutated NSCLC [16]. Negrao et al. observed that trametinib with or without dabrafenib or lifirafenib had a more potent inhibition of BRAF non-V600 mutant NSCLC cell lines than other MEK, RAF, and ERK inhibitors when compared to the inhibition of V600 mutants [14]. Negrao et al. also suggested that, while there are many unknown variants of the non-V600 BRAF mutant cancers, assessing for high clonality in addition to the class of the mutation will allow for identification of variants that are more likely to be oncogenic drivers and thus be more responsive to RAF/MEK therapy [14]. Our case of an extremely rare variant of a non-V600 BRAF mutant NSCLC also represented positive response to RAF/MEK inhibitors, but more research needs to be done to better assess RAF/MEK inhibitor’s activity in treatment of non-V600 mutations.

Targeted treatment strategy specifically for patients with non-V600E mutated NSCLC has not been established yet. Miyauchi et al. investigated a newly discovered pan-RAF inhibitor, LY3009120, which has demonstrated efficacy in treating cancers with various BRAF genotypes, including NSCLC with non-V600 mutation [2]. They concluded this agent could present potential new therapy for patients with non-V600 BRAF mutation if they fail to respond to RAF/MEK inhibitors [2].

## 4. Conclusions

Treatment of cancers harboring a non-V600 mutations is challenging due to their functional heterogeneity, lack of knowledge of their clinical significance, or established target therapies. Categorizing BRAF mutations into three classes and degree of clonality helps predict a treatment response for a given BRAF mutation, but more research needs to be done to better understand different variants, especially of the non-V600 type. We presented an extremely rare variant of a non-V600 BRAF (specifically p.T599dup) mutant NSCLC that responded well to RAF/MEK inhibitor combination, which is usually reserved for class 1 mutations, predominantly V600. By presenting this case, we hope to bring more attention to rare non-V600 BRAF mutations. Non-V600 BRAF-mutated NSCLC tends to have a more aggressive clinical behavior and resistance to chemotherapy, which is why it is so important to continue classifying non-V600 BRAF mutants and their response to therapies. While some studies have shown that RAF/MEK therapy can be effective in non-V600E BRAF mutations, given the difference of the two mutations, there may be a better targeted immunotherapy for non-V600E mutant NSCLC. More research needs to be done to investigate this. A newly discovered pan-RAF inhibitor, LY3009120, DIF inhibitors, as well as immune checkpoint inhibitors need to be further investigated as a potential new therapy for non-V600E BRAF mutations.

## 5. Clinical Practice Points

- Majority of BRAF in NSCLC are of non-V600 type;
- Some of them have shown response to V600 targeted therapies;
- More research needs to be done to better understand the non-V600 mutations and new targets for non-V600 therapies.

## Data Availability

Data is contained within the article or supplementary material.
